# Medical Ethnobotany of the Bissau-Guinean Community of Migrants Living in Northern Italy and Comparison with the Ethnopharmacology of Guinea-Bissau

**DOI:** 10.3390/plants12091909

**Published:** 2023-05-08

**Authors:** Alfredo Sambù, Laura Cornara, Luís Catarino, Bucar Indjai, Marco Biagi, Paolo Giordani

**Affiliations:** 1 Department of Physical, Earth and Environmental Sciences, University of Siena Strada Laterina 8, 53100 Siena, Italy; festawi@hotmail.com (A.S.); biagi4@unisi.it (M.B.); 2 Department of Earth, Environment and Life Sciences, University of Genova, 16132 Genova, Italy; 3 Centre for Ecology, Evolution and Environmental Changes (cE3c) & CHANGE—Global Change and Sustainability Institute, Faculdade de Ciências, Universidade de Lisboa, 1749-016 Lisbon, Portugal; lmcatarino@fc.ul.pt; 4 INEP—Instituto Nacional de Estudos e Pesquisa, C.P. 112 Bissau, Guinea-Bissau; indjai.b@gmail.com; 5 CEF—Centro de Estudos Florestais, Instituto Superior de Agronomia (ISA), Universidade de Lisboa, 1349-017 Lisboa, Portugal; 6 Department of Pharmacy, University of Genova, 16148 Genova, Italy; giordani@difar.unige.it

**Keywords:** traditional ethnobotanical knowledge, West Africa, migrants, medicinal plants, ethnic groups, interrelations between plants and peoples

## Abstract

This study compares the knowledge of medicinal plants of Bissau-Guinean migrants now established in Italy with the ethnopharmacology still present in their country of origin. We also investigated how traditional ethnobotanical knowledge is changing following the phenomenon of migration from Africa to Europe. The ethnobotanical data were collected during 2017–2018, by interviewing 49 informants belonging to 8 ethnic groups, living in 8 provinces of northern Italy. The final inventory of botanical taxa included 81 species belonging to 34 families, with Fabaceae and Malvaceae the most represented, followed by Euphorbiaceae, Apocynaceae, Combretaceae, and Solanaceae. Plant remedies were used to treat 21 ailment categories, such as fever, internal infections, intestinal and respiratory problems, and pains. The traditional ethnobotanical knowledge of Bissau-Guinean migrants in Italy was associated with gender, with women showing the highest knowledge. In addition, a negative relationship was observed between the maintenance of this knowledge and the number of years migrants have spent in Italy. Overall, a loss of knowledge was observed in the less numerous ethnic groups. However, traditional preparations based on plants from the country of origin are in general well preserved to maintain a good state of health. Our work could help in transferring to the next generation the cultural heritage of Bissau-Guinean people permanently moved to European Countries.

## 1. Introduction

Over the last few decades dynamics of traditional plant knowledge and practices have been also investigated in the context of the large increase in the scale of the global migration [1]. Intercontinental migration from Africa is largely directed towards Europe which remains by far the major destination of sub-Saharan migrants. In 2000, the number of sub-Saharan African migrants living in Europe was close to 3 million [2]. In addition, migration from Sub-Saharan Africa has grown dramatically since 2010 [3].

Since the seventies, there has been a migration of people from Guinea-Bissau to northern Italy, as a result of the strong link between the people of this African Country and the Italian Catholic Church, following the election of Mons. Settimio A. Ferrazzetta, a Franciscan native to Verona, as the first bishop and primate of Guinea-Bissau. The migrant community of Guinea-Bissau established in Italy includes 2326 persons (2123 male, 203 female), mainly living in northern Italy [4], and belonging to different ethnic groups (Balanta, Bijagos, Felupe, Fula, Mancanha, Mandinga, Mandjaco, and Pepel). These migrants have been progressively involved in the social context of Italian cities but, at the same time, they have brought with them their customs and traditions, and the remains of this wealth of knowledge have not yet been considered. To date, only a few ethnobotanical studies have evaluated the effects on the cultural heritage of traditional ethnobotanical knowledge of migrants living in Italy, following the encounter with a culture very different from that of origin [5,6,7,8]. In addition, the socio-cultural context and the conceptualization of the processes of health/illness of migrants has been poorly considered. In general, for a high percentage of the population of Guinea-Bissau a holistic and systemic vision of the universe persists, where animals and plants are seen as elements of the world at the same level as humans [9].

In the migrant community, specific botanicals that are culturally important in their African country are considered identity-bound and used also in the host countries. Ethnobotanical investigation can be an effective tool to evaluate how much of the wealth of folk medicine remains in a migrant people following their displacement to a new country. This kind of study must take into account the occurrence of transnational social and trade networks between the migrants and their home country. The poor availability of herbs and food plant items in food shops in the host country, due to strict importation laws [10], often causes improper importation of these products by migrants and their relatives/friends from the country of origin to Italy.

In the literature, scientific works about the traditional ethnobotanical knowledge of the Bissau-Guinean community in Italy are not available, while recent works on medicinal plants of Guinea-Bissau have been published [11,12]. Therefore, the purpose of this study is to compare the knowledge of medicinal plants of Bissau-Guinean migrants now established in northern Italy with the ethnopharmacology still present in Guinea-Bissau. In addition, we investigate if and how this knowledge is changing following the phenomenon of migration from Africa to Europe. These data are of crucial importance to keep the focus on the integration of African migrants into host European countries.

## 2. Results

### 2.1. The Medical Ethnobotany of the Bissau-Guinean Migrants in Northern Italy

Informants, with a mean age of 41 years, belong to the first generation of Bissau-Guinean migrants and to 8 ethnic groups, with the Balanta group being the most represented, followed by Pepel. Participants were selected using the snowball sampling method among the Bissau-Guinean migrant communities of northern Italy.

In Table 1 our data are summarized, showing that the final inventory of botanical *taxa* included a total of 81 species belonging to 34 plant families. Fabaceae (20%) and Malvaceae (7.1%) are the most represented families, followed by Euphorbiaceae (5.9%), Apocynaceae, Combretaceae, and Solanaceae (4.7%). In addition, other ethnomedicinal remedies were also reported, such as python and snake fat; mushroom; fish; slime; or mud. In some cases (10 *taxa*), it was not possible to identify the species based on the vernacular name referred to by informants nor by comparison with bibliographic sources or images from scientific texts or online databases.

**Figure 1:**
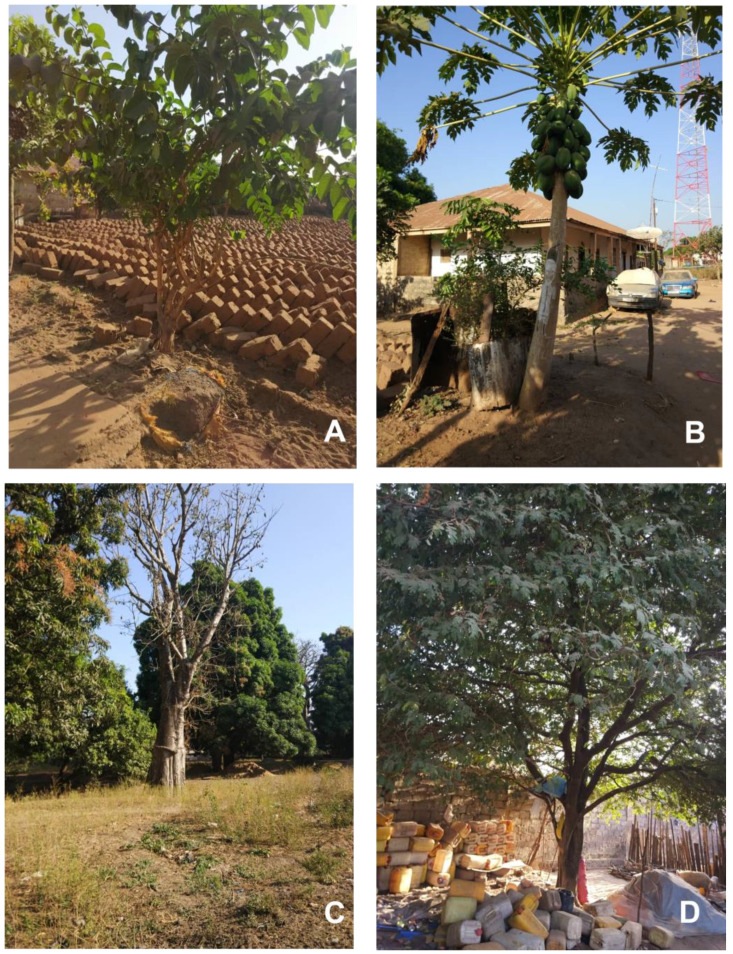
Useful trees around the village of Matandin (Prabis Municipality): (**A**) *Sarcocephalus latifolius*, madronha; (**B**) *Carica papaya*, papaya; (**C**) *Adansonia digitata*, cabacera; (**D**) *Tamarindus indica*, tambarina. Photos from J. Sambù Bambaia and Domingos Sambù.

Among the most important medicinal plants were trees and shrubs (Figure 1A–D). The highest RFCs (Table 2) were found for *Sarcocephaus latifolius* (0.29) (Figure 1A), followed by *Senna occidentalis* (0.27), *Carica papaya* (0.25) (Figure 1B), and *Jatropha curcas* (0.22). Among cultivated plants, also *Adansonia digitata* (Figure 1C), *Mangifera indica*, *Anacardium occidentale*, *Musa* × *paradisiaca*, *Psidium guajava*, *Tamarindus indica* (Figure 1D), and *Citrus aurantifolia* were frequently used (RFCs > 0.1).

**Figure 2:**
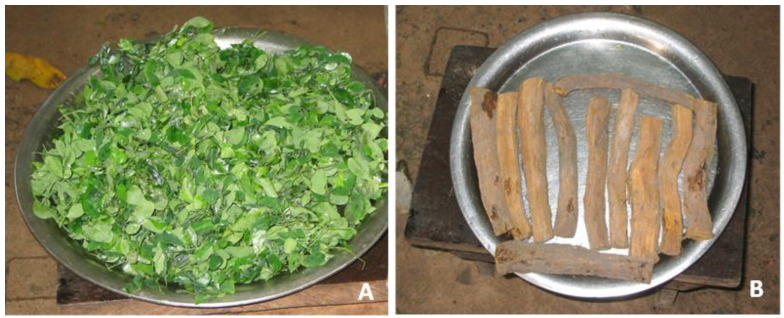
Examples of plants frequently used by migrants in Italy. (**A**) *Moringa oleifera*, “nené badadji”, fresh leaves. (**B**) *Sarcocephalus latifolius*,”madronha”, roots and bark. Photos from L. A. Vieira Gomes.

**Figure 3:**
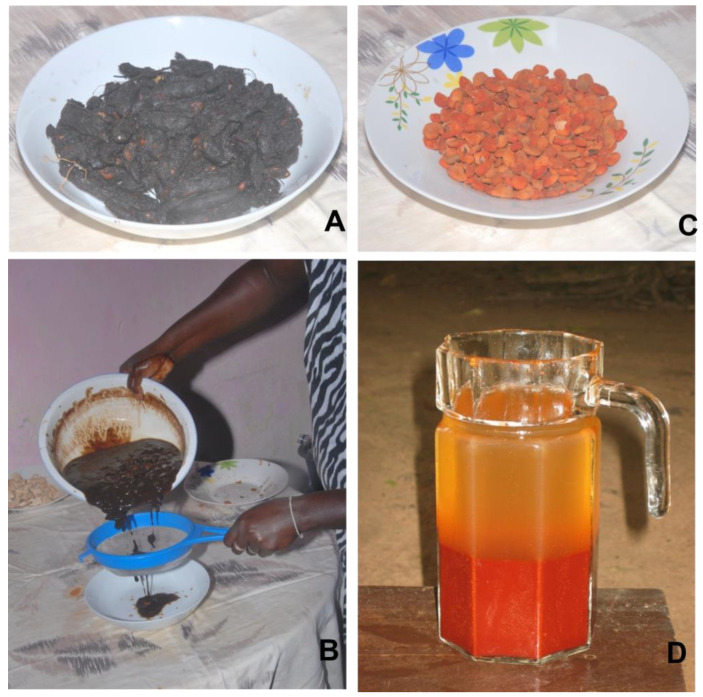
Fruits of *Tamarindus indica*, “tambarina” (**A**) macerated in water, pressed, and filtered (**B**) to obtain a juice indicated as febrifuge. Seeds of *Dialium guineense*, “veludo” (**C**); the juice obtained by maceration in water and pressing (**D**) is drunk as a tonic. Photos from L. A. Vieira Gomes (Nené Matcho) of Mrs. S. Oliveira Omena during juice preparation, and from M. Djalo and Dr. F. Biag (Kanda) (Bissau, Ponta Neto/Bor-Comune di Prabis).

**Figure 4:**
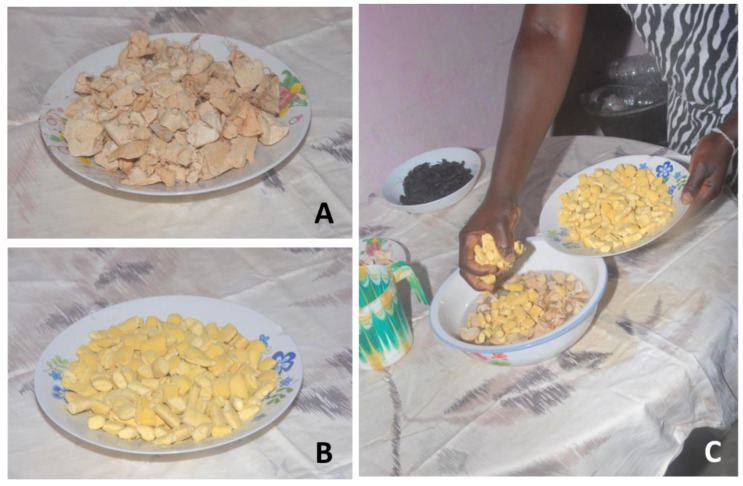
Fruits of *Adansonia digitata*, “cabacera” (**A**) and *Parkia biglobosa*, “foroba” (**B**) mixed and macerated in water for a few minutes (**C**) to obtain a juice that is filtered and given as a tonic and invigorating to children. Photos from L. A. Vieira Gomes (Nené Matcho) of Mrs. S. Oliveira Omena during preparation of juice, with supervision of D. Gomes da Silva (Bissau, Cuntum Madina).

The most used parts of the plants were leaves (from 57 species), followed by seeds (18) and fruits (16), bark (15), and roots (14) (Figure 2, Figure 3 and Figure 4). Principal methods of herbal preparations included decoction-infusion (53%), maceration (23%), chewed (5%), oil (3%), and others (about 6%). 

Plant remedies were used to treat 21 ailment categories, mostly in agreement with Catarino et al. [11]. The greatest number of plants were used to treat various infections (INF), both internal (INFI) or external (INFE), fever (F), intestinal problems (C), pains (A), and cough and respiratory diseases (E).

Table 3 shows the plants and uses mainly maintained by the majority of informants who live permanently in Italy. We have, in fact, observed that informants remain attached to their cultural beliefs and traditions, even after settling in the new country. Therefore, they preferred to continue using medicinal plants from their country of origin, because of a greater trust in traditional medicinal remedies compared to pharmaceutical products and food supplements commercially available in the host country. Furthermore, we have found that they have more confidence in the remedies they prepare themselves, following traditional recipes, because they are convinced that this can increase their healing power.

The availability of herbal material is made possible by the dense network of relationships with relatives and friends who guarantee the supply of such material. Only in rare cases have the migrants tried to cultivate some medicinal plants in northern Italy that they used in the native country, e.g., *Abelmoscus esculentus* cultivated during the summer in allotment gardens near the homes.

Among the most frequently used remedies, fresh leaves of *Moringa oleifera* (Figure 2A) were macerated and used in case of conjunctivitis. An infusion of dried powdered leaves of this species was also indicated to treat hypertension, arrhythmia, and palpitations, while leaf decoction was used to wash the body in case of yellow fever. In addition, leaves pounded and boiled together with the peanut sauce were an ingredient of the course called “badadji/candja” and were generally mixed with food for their invigorating effects.

Roots of *Sarcocephalus latifolius* (Figure 2B) were macerated in water and the decoction was used against different diseases, such as pains, intestinal problems, blood disorders, and others.

Fruits and seeds of *Tamarindus indica*, “tambarina”, were macerated in water, then pressed and filtered to obtain a juice (Figure 3A,B) indicated for its febrifuge properties. In addition, the juice obtained from seeds of different species by maceration in water and filtering was used as a tonic and invigorating for both adults and children, e.g., seeds of *Dialium guineense,* called “veludo” (Figure 3C,D) and seeds of *Adansonia digitata*, “cabacera” (Figure 4A) mixed with those of *Parkia biglobosa*, “foroba” (Figure 4B,C).

*P. biglobosa* and *D. guineense* are widely used as an important source of nutrition for West African populations for their high protein, vitamin, and micronutrient contents. However, these species are also quoted for their medicinal properties, such as *P. biglobosa* used as a cardiovascular protective and for its anti-diabetic effects, and *D. guineense* to prevent schistosomiasis [13] and references therein. In fact, apart from their dietary benefits, our informant cited seeds of *P. biglobosa* also as beneficial against yellow fever, typhus, and malaria, seeds of *D. guineense* as antimalarial, and the leaves and fruit of *A. digitata* for their immunostimulant effect.

### 2.2. Comparative Analyses of the Ethnobotanical Traditional Knowledge

**Figure 5:**
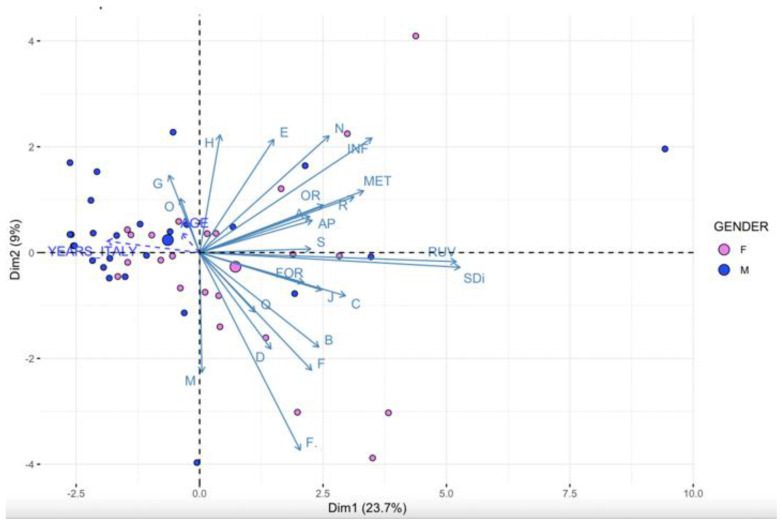
Representation of the first two factors of a Principal Components Analysis (PCA) in relation to TEK (SD, RUV, and specific medicinal categories) and to social characteristics of informants (age, gender, ethnic group and time spent in Italy). Codes of the disease categories are reported in Table 1.

Although the overall variance explained by the first two components of the PCA is rather low (ca. 20%) (Figure 5), a clear positive relationship can be observed between the first component (PC1) and an overall knowledge gradient, described by increasing values of SDi and RUV (squared loadings, respectively, 0.73 and 0.66). Moreover, the ethnobotanical knowledge of Bissau-Guinean migrants in Italy was associated with gender (squared loadings = 0.43), with women showing the highest knowledge. It is interesting to note that there is also a negative relationship between ethnobotanical knowledge and the age of the informants (squared loadings = 0.1) and the number of years they have spent in Italy away from their country of origin (squared loadings = 0.3). These findings could be related to the dynamic of migration: men moved first to Italy and usually spent some years alone, before family reunification. On the other hand, the relationships with other variables describing the social structure of the migrant community are weakly correlated with overall botanical knowledge (PC1). In addition, they are only partially related to some particular use categories along the second component of the PCA (PC2). For example, ethnic group was also a source of variation (squared loadings on PC2 = 0.52), being mainly associated with, e.g., skin inflammation and heart diseases mainly reported by Mancanha informants (Figure 5).

**Figure 6:**
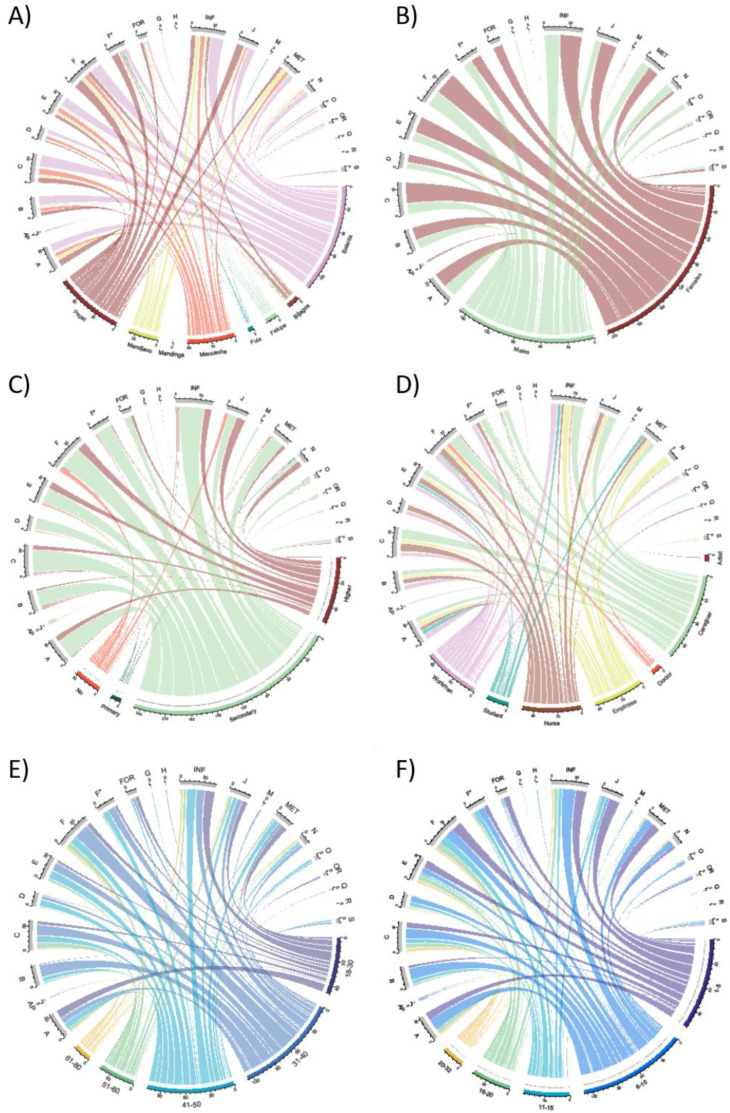
Circular plots showing the relationships among referred disease categories and the informants grouped based on different social factors: (**A**) ethnic group; (**B**) gender; (**C**) education; (**D**) job; (**E**) age; and (**F**) years since their arrival in Italy. Color bands summarize relationships between each disease category and population group. Numbers below the external segments in the graphs represent the total numbers of citations for each disease category and population group. Codes of the disease categories are reported in Table 1.

The diagrams in Figure 6 report the most important associations between informant groups and disease categories. The ethnic groups most represented among the informants (esp. Balanta) were also those with the highest number of overall reports and the widest variety of disease categories reported (Figure 6A). As far as gender is concerned (Figure 6B), females reported more uses overall than males, although most categories were reported equally by both genders. Some exceptions can be observed for categories J, FOR and E (mainly reported by females) and categories N and O (predominantly reported by males). Most of the informants had a secondary level of education and, consequently, this group is the one reporting the most use. Those with higher education, on the other hand, are not necessarily those with more ethnobotanical knowledge, so much so that they report no use for several categories, e.g., O, OR, Q, and R (Figure 6C). Category D (skin inflammation) was mainly reported by workmen and nurses, while doctors overall reported fewer ethnobotanical uses focusing on the treatment of fever (F) and infections (INF). For other disease categories, on the other hand, a rather even distribution of reports by the different job groups is observed (Figure 6D). Interestingly, older migrants are not those who report more ethnobotanical information, which is instead associated with the middle or even younger classes (Figure 6E). This finding could be related to the period since the migration to Italy (Figure 6F). In particular, some categories of use are almost exclusively reported by informants who have been in Italy for less than 10 years: for example, species used for the treatment of metabolic disorders (MET), pain relief (A), anemia and other blood disorders (J), and infections (INF).

The results of the comparison of ethnobotanical functional uses in the migrant Bissau-Guinean community in Italy and the healers interviewed in the country of origin by [11] are presented in Table 4. The comparison is reported in terms of Jaccard dissimilarity so that the higher the value in the table, the greater the differences in species use between the groups of informants compared. Considering the entire migrant Italian community, a considerable level of overlap of their ethnobotanical knowledge with that of the healers in Guinea-Bissau is observed (Jaccard dissimilarity = 0.10). A comparable value of Jaccard dissimilarity (0.11) is also observed in the case of the most numerous ethnic groups among migrants in Italy, the Balanta, who also share much of the ethnobotanical information with the other ethnic groups in Italy (Jaccard dissimilarity = 0.11). In general, as might be expected, larger groups showed more overlapping knowledge than ethnic groups represented by few informants. As such, Felupe, Bijagos, and Fula, with less than 5 people interviewed among migrants in Italy, have a Jaccard dissimilarity > 0.5 of reported ethnobotanical uses. This is confirmed both with respect to the rest of the Italian community and to the overall knowledge in Guinea-Bissau, but also with respect to the knowledge of healers of their own ethnic group in the country of origin.

Overall, migrants from Guinea-Bissau maintain a strong link with the traditional use of plants typical of their country of origin. In some of the less numerous ethnic groups among migrants there is a fragmentation of knowledge. This is shown by a lower number of species, although in some cases a small set of species was cited only in Italy and not reported by informants in Guinea-Bissau. For example, the flowers of *Sesamum radiatum* for an anti-lice foamy emulsion, the leaves and root of *Acanthospermum hispidum* as a diuretic, and the leaves and flowers of *Abelmoschus sabdariffa* for metabolic diseases. The red color that characterizes the infusion of these flowers is associated with blood and for this reason, it is believed that this remedy is able to purify the blood and increase its iron content.

In other cases, in the absence of the availability of species normally found in Guinea-Bissau, some migrants in Italy used similar species for medicinal purposes. For example, they cited the use of *Mentha* sp., *Allium* sp., *Laurus nobilis,* and *Citrus* spp., whose utilization was probably influenced by the cultural contamination in the host country. However, these common medicinal plants, which have now spread almost worldwide, are often used in particular ways, influenced by the cultural traditions of the native country. For example, informants have reported using the decoction of *Eucalyptus* leaves to treat respiratory ailments, initially by suffumigation and then to wash the body, in the belief that it is also useful for lowering fever. Similar uses have been referred to for the leaves of *Laurus nobilis* and *Citrus* sp.

### 2.3. Other Remedies and Unusual Uses

The use of *A. senegalensis*, *T. macroptera*, and *C. papaya* has been reported by our informants as a practice used during pregnancy, childbirth, and breastfeeding of newborns.

Python (piton) and *Naja* snake (bida) fat is extracted from reptiles. It is a solid fat that if heated becomes a pale-yellow oil. One informant referred to the use of python oil to treat pains, while another informant reported the antiviral properties of cobra fat against the *Herpes* virus. 

The use of tripe of the fish *Tilapia guineensis* has been indicated by one informant as a method to stop the vomiting of drunk people. Another informant referred to the fact that cow hooves are pulverized, and the powder can be boiled with water and sugar to obtain an antitussive syrup. An unidentified alga growing near fountains is used to treat chronic wounds, while an unidentified mushroom (*cugumelo*) is quoted to relieve pain in the feet, and also to treat fungal skin infections (“*fungus against fungus*”). The river mud is used as a wrap in the case of a sprained ankle, while water and salt are used to treat blisters and calluses.

Two informants reported a particular ancient custom performed by the ethnic group of Balanta, called *n’masn n’betn,* meaning treatment for “dog’s disease”, consisting of sadness or swelling of the belly or face. This remedy consists of preparing a soup mixing seed of *fundo* (corresponding to the food plant *Digitaria exilis* (Kippist) Stapf) with the leaf of a plant probably belonging to the genus *Psychotria* (Rubiaceae). 

## 3. Discussion

As a major result, we found that the ethnobotanical knowledge of Bissau-Guinean migrants in Italy was associated with some social characteristics of the community. First, gender played a key role, with women showing the highest knowledge. On the contrary, there was a negative relationship between ethnobotanical traditional knowledge vs. the age of the informants and the number of years the migrants have spent in Italy away from their country of origin. These findings could be related to the dynamic of migration: men moved first to Italy and usually spent some years alone, before family reunification. Additionally, older people were often those who migrated earlier, and it is possible that they have changed their care habits, forgetting, or neglecting the traditional medicine of Guinea-Bissau. In particular, informants who have been in Italy for less than 10 years almost exclusively reported some categories of use: for example, species used for the treatment of metabolic disorders (MET), pain relief (A), anemia and other blood disorders (J), and infections (INF).

Overall, migrants from Guinea-Bissau, even after settling permanently in Italy, maintain a strong link with the traditional use of plants typical of their country of origin, particularly about preparations useful to maintain a good state of health, considered as supplements to the diet. This situation might be due to the maintenance of a strong network of relationships among them, which guarantees the supply of herbal material. This is generally transported by the migrants themselves, after having gone to Guinea-Bissau to visit their families. Therefore, the knowledge about folk medicine is mostly conserved in the whole community, although a loss effect related to a higher number of years spent in Italy is observable. Particularly, this effect is exacerbated in some of the less numerous ethnic groups, representing a potentially negative effect on the transferring of cultural heritage to the next generations. Interestingly, in some cases, there was a small set of tropical species cited only in Italy but not reported by informants in Guinea-Bissau [11]. For example, the flowers of *Sesamum radiatum* to obtain an anti-lice foamy emulsion, the leaves and flowers of *Hibiscus sabdariffa* for metabolic problems, and the leaves and root of *Acanthospermum hispidum* as a diuretic. In other cases, we observed a substitution process according to which, in the absence of the availability of species normally found in Guinea-Bissau, some Italian migrants used *Mentha* sp. and *Allium* sp. for metabolic diseases or *Laurus nobilis* for digestive problems. Such uses could be related to cultural contamination in the host country.

Some ethnobiological studies on Guinea-Bissau traditions were carried out towards the end of the 1950s by the Italian anthropologist Prof. Antonio Scarpa (1903–2000). Concerning the traditional use of plants [14], he described the «lactatio agravidica» (breastfeeding without pregnancy), pointing out the use of several galactagogue plants to induce or improve lactation. In a case report from the Gabù district, this effect was obtained with oral administration of *Terminalia macroptera* and *Annona senegalensis* [15]. Among the galactagogue plants, he also reported the mature fruit of *Carica papaya* widely used by Bijagos, Felupe, and Baiot, while other tribes introduced dried and pulverized queen termite into food for the same purpose [16]. Analogous use of *A. senegalensis*, *T. macroptera*, and *C. papaya* is a practice still in use today during pregnancy, childbirth, and breastfeeding of newborns. 

Some ancient customs are still known among our informants, such as the python fat traditionally used for the treatment of rheumatism, boils, keloids, and broken bones in Nigeria [17,18]. In some cases, the use of the seeds of fundo (*Digitaria exilis*), together with plant leaves (probably *Psychotria* sp., Rubiaceae), as a treatment of sadness, swelling of the belly or face, occurring after having trampled human blood or after being witnesses of someone’s death, was reported. It can be assumed that this observation refers to *Psychotria peduncularis* (Salisb.) Steyerm, a well-known African shrub also reported in the survey of Catarino et al. [11] for the treatment of different diseases, and, in particular, mental and neurological disorders. Accordingly, it was also cited by Romeiras et al. [19] as a remedy to treat “bad spirits and mandjidura” (witchcraft).

## 4. Materials and Methods

### 4.1. Study Area and Fieldwork

#### 4.1.1. Northern Italy and Ethnic Diversity 

The ethnobotanical data were gathered during 2017–2018 through extensive dialogues and semi-structured interviews with 49 informants (26 male, 23 female), ranging from 18 to 80 years old, with a mean age of 41 years. Informants live in 8 Italian provinces (Verona, Vicenza, Bologna, Treviso, Brescia, Lecco, Trento, and Padova) and belong to 8 ethnic groups: Balanta (21 informants), Bijagos (3), Felupe (3), Fula (1), Mancanha (6), Mandinga (1), Mandjaco (5), and Pepel (9). Participants in northern Italy were selected using the snowball sampling method among the first generation of Bissau-Guinean migrants. For every informant, we recorded personal information (age, gender, education level, profession, and population group) to explore differences and similarities between citations based on different factors. The informants had ethnobotanical knowledge because of family tradition and personal experience in self-medication using herbs. In these interviews, the informants were requested to indicate vernacular names of plants, parts of the plant used, uses, and preparation procedures, including information on specific recipes. In most cases, samples of plants used as remedies were shown by informants making it possible to verify the plant taxa. In fact, the co-authors native from Guinea-Bissau, having specific knowledge of the local medicinal flora, were able to confirm the correct identification of the species even starting from the plant portion used. However, when the plant portion was not available, images taken from scientific texts [20,21], and the online database West Africa Plants—A photo guide [22], were shown to informants asking them to indicate the plant used as a remedy. Interviews were conducted in Creole or Italian. Prior Informed Consent (PIC) was obtained verbally before commencing each interview; interviews were conducted according to the ethical guidelines of the International Society of Ethnobiology Code of Ethics [23]. Plant names have been checked and updated with the “Plants of the world online” database (www.powo.science.kew.org) provided by the Royal Botanic Gardens (Kew), in accordance with the survey carried out in Guinea-Bissau by Catarino et al. [11].

#### 4.1.2. Guinea-Bissau and Ethnic Diversity 

Guinea-Bissau (36,125 km^2^) located in West Africa and wedged between Senegal, the Republic of Guinea, and the Atlantic Ocean0 has a total population of about 2,000,000 (https://www.worldometers.info/world-population/guinea-bissau-population/ accessed on 17 February 2022), with the highest density in the North Western areas. The capital Bissau, located in the central coastal region, accounts for about 25% of the country’s total population and is the preferred destination of internal migrants. Guinea-Bissau’s territory includes different zones characterized by various types of vegetation as previously described by Havik and Daveau [24] and Catarino et al. [11]. Due to the progressive sahelisation, humid forested areas are receding, and the dryer savanna-type vegetation is advancing, threatening living conditions and economic development [25]. In addition, the biodiversity of the vascular flora, estimated to encompass 1507 species, of which 1459 are native, is at risk [25]. 

A complex mosaic of more than thirty ethnic groups is present, with Fula (28.5%), Balanta (22.5%), Mandinga (14.7%), Pepel (9.1%) and Mandjaco (8.3%) being the most represented. Fula, Mandinga, and a few smaller groups (Biafada, 3.5%; Balanta-Mané,1%; Nalú, 0.9%; Sarakolé, 0.5%) are wholly or partly Islamised. On the contrary, the Balanta, Pepel, Mandjaco, Mancanha (3.1%), Bijagós (2.1%), and Felupe/Jola (1.7%), living in littoral regions, have been converted to Catholicism or practice African religions. Actually, the previous cosmological vision in a syncretic form remains very widespread according to which supernatural beings protect people and the territory [9]. In addition, part of the population practices more than one religion and almost 16% does not indicate adherence to a particular religious practice [11]. Most of the population (90.4%) speaks Guinean Creole (GC) or Kriol, but a variant of GC is spoken in the Lower Casamance region in Southern Senegal. However, the official language is Portuguese, spoken by over a quarter of the population.

#### 4.1.3. Quantitative Analysis

Quantitative analyses of data were performed by ethnobotanical indices:

- Relative Use Value (RUVi)

RUVi = [(Σ UVis/UVs)]/n;(1)

where UVis = number of uses that informant i knows for species s, UVs = use value of species s (=average number of uses that informant know for species s), and n = number of useful species.

This index measures how many plants uses an informant knows relative to the average knowledge among all informants [26].

- Relative Frequency of Citation (RFC) is calculated as follows:

RFC = FC/N (0 < RFC < 1).(2)

This index shows the local importance of each species, and it is given by the frequency of citation. FC is the number of informants mentioning the use of the species and N is the total number of informants participating in the survey, without considering the use categories [27].

### 4.2. Statistical Analyses

The ethnobotanical data collected from northern Italy and Guinea-Bissau were compared with each other. The pre-existing literature on the traditional pharmacopeia of Guinea-Bissau was used [11,12].

In order to visualize the information contained in the data sets, statistical analyses were carried out in the R environment (vers. 3.6.3, R Core Team 2020). Principal component analysis (PCA) was performed on autoscaled data using the ‘FactoMineR’ package. The relationships between groups of informants and categories of use mentioned were summarised by circular plots, using the circlize package (vers. 0.4-2 [28]).

Following Villéger et al. [29,30], we calculated the ethnobotanical functional richness of each community (‘Guinea-Bissau’ and ‘Italy’ dataset) as the amount of the functional space filled by each cited remedy. The functional richness is measured using the volume inside the convex hull, which encloses all the remedies reported by each community.

We calculated the functional dissimilarity (Fb) between the Bissau-Guinean communities in Italy and in the original country as the ratio between the volume in the functional space not shared by the two communities relative to their total functional diversity (total volume).

## 5. Conclusions

Our data showed a well-defined picture according to which the Bissau-Guinean community in Italy preserves most of the ethnobotanical knowledge of the country of origin, probably thanks to the maintenance of a strong social network of relationships among migrants and with relatives still living in Guinea-Bissau. The loss of knowledge related to a higher number of years spent in Italy was more evident in the less numerous ethnic groups among migrants. This represents a potential threat to the transmission of own ethnobotanical traditions to the next generations living in the host country. Our survey could help in maintaining and transferring the cultural heritage of Bissau-Guinean people permanently moved to European Countries.

## Figures and Tables

**Table 1:** Medicinal plants used by Bissau-Guinean migrants in northern Italy and comparison with the use of the same plants in the country of origin according to Catarino et al. [11].

Species	Family	Vernacular Names	Parts Used	Preparation	Use	Group of Diseases	No. Citations Per Population Group	Catarino et al. [11].	Other Uses
*Allium sativum* L.	Amaryllidaceae	adjo, alho	Bulb	Maceration	I	MET, OR	2 (Pepel)		Food
*Anacardium occidentale* L.	Anacardiaceae	cadju	Bark, Leaves, Seeds	Decoction	I/E	A, O, OR, MET	5 (2 Pepel, 1 Balanta, 1 Felupe, 1 Mandjaco)	X	Food
*Mangifera indica* L.	Anacardiaceae	mango di terra	Bark, Leaves	Decoction, Maceration	I/E	A, E, INFI, MET	5 (2 Mandjaco, 2 Balanta, 1 Mancanha)	X	Food
*Spondias mombin* L.	Anacardiaceae	mandiple	Fruits	Maceration	I	C	1 (Balanta)	X	Food
*Annona muricata* L.	Annonaceae	pinha	Leaves	Decoction	I	A, C	3 (2 Mancanha, 1 Mandjaco)	X	Food
*Annona senegalensis* Pers	Annonaceae	bodi iodi	Leaves	Decoction	E	O	1 (Balanta)	X	Food, Vet
*Uvaria chamae* P. Beauv.	Annonaceae	banana santchu	Leaves	Decoction	I/E	F, F*	2 (1 Mancanha, 1 Mandjaco)	X	Food
*Alstonia congensis* Enl.	Apocynaceae	bantamforo	Roots	Decoction	I	F, INFI	2 (Pepel)	X	
*Cryptolepis sanguinolenta* (Lindl.) Schltr.	Apocynaceae	cuntes	Leaves, Roots	Maceration	I	INFI	2 (1 Balanta, 1 Mancanha)	X	
*Holarrhena floribunda* (G.Don) T. Durand and Schinz	Apocynaceae	rubitche, brigpatch, cola di terra, bukonu mabo, po-de-pinti	Leaves, Seeds	Decoction, Maceration	I/E	A, E, INFE, OR	4 (3 Balanta, 1 Felupe)	X	
*Landolphia dulcis* (R.Br. ex Sabine) Pichon	Apocynaceae	fole, mancubar	Fruits	Juice, Maceration	I	FOR, J	2 (Pepel)	X	Food
*Colocasia esculenta* (L.) Schott	Araceae	manfafa	Leaves	Decoction	I		1 (Balanta)		Food
*Elaeis guineensis* Jacq	Arecaceae	palmera, citi, siti bidado	Fruits, Seeds	Decoction, Oil	I/E	A, F, INFI	12 (4 Balanta, 4 Pepel, 2 Bijagos, 1 Mancanha, 1 Mandjaco)	X	Food, Cosm
*Acanthospermum hispidum* DC.	Asteraceae	nhara sikido	Leaves	Decoction	I	J, N	2 (Balanta)		
*Lactuca sativa* L.	Asteraceae	alfas	Leaves	Decoction	I	J, MET, N	4 (2 Balanta, 2 Pepel)		Food
*Sphaeranthus senegalensis DC.*	Asteraceae	dockn mponkda, m’thobethobe	Leaves	Decoction	I	B	1 (Balanta)		
*Crescentia cujete* L.	Bignoniaceae	cabas	Leaves	Decoction	I	F	1 (Pepel)		
*Carica papaya* L.	Caricaceae	papaia	Fruits, Leaves, Roots, Seeds	Decoction, Ground	I/E	AP, C, F, F*, FOR, INFI	17 (7 Balanta, 4 Pepel, 2 Bijagos, 1 Felupe, 1 Fula 1 Mancanha, 1 Mandjaco)	X	Food
*Neocarya macrophyla* (Sabine) Prance ex F. White	Chrysobalanaceae	tambacumba	Leaves, Roots, Seeds	Decoction	I	FOR, INFI, MET	3 (2 Mandjaco, 1 Balanta)	X	Food
*Combretum adenogonium* Steud. ex A.Rich.	Combretaceae	djambacatam	Leaves	Decoction	I	N	1 (Mandinga)	X	
*Combretum micranthum* G.Don	Combretaceae	quemkeleba, sanfito, psangla, buck, buco, café bravo, cha de buco	Branches, Leaves	Chewed, Decoction, Infusion, Maceration	I/E	A, B, C, E, F, FOR, J, MET, N	11 (4 Balanta, 2 Felupe, 2 Mancanha, 2 Mandjaco, 1 Pepel)	X	
*Guiera senegalensis* J.F.Gmel.	Combretaceae	iuci, badodos	Branches, Leaves	Chewed, Decoction	I/E	A, B, C	6 (4 Balanta, 1 Mandjaco, 1 Pepel)	X	Cosm
*Terminalia macroptera* Guill. and Perr.	Combretaceae	massite, untulaa’, untulam	Leaves, Roots, Seeds	Decoction, Maceration, Oil	I	A, B, F, F*, FOR, INFI, J, M, N	13 (6 Pepel, 3, Balanta, 3 Mandjaco, 1 Fula)	X	
*Calycobolus heudelotti* (Baker ex Oliv.) Heine	Convolvulaceae	m’mas mdam, massemedam	Leaves	Ground, Maceration	I/E	A, E, F	4 (Balanta)	X	
*Ipomoea asarifolia* (Desr.)Roem. and Schult.	Convolvulaceae	lacacon, n’tumbuli	Branches, Leaves	Decoction	I/E	B, E	4 (2 Balanta; 1 Mandjaco, 1 Pepel)	X	
*Cucurbita pepo* L.	Cucurbitaceae	abobra	Seeds	Ground, Maceration	I	B, C	2 (Balanta)		Food
*Luffa cilindrica* (L.) M. Roem	Cucurbitaceae	djadar	Fruits, Leaves	Decoction	I	A, N	2 (Balanta)	X	
*Alchornea cordifolia* (Schumach. and Thonn.) Müll. Arg.	Euphorbiaceae	blore	Latex, Seeds	Extraction, Oil	I/E	H	2 (Balanta)	X	Cosm
*Anthostema senegalese* A. Juss.	Euphorbiaceae	ptom	Branches, Leaves	Ground	I	G	1 (Balanta)	X	
*Hymenocardia acida* Tul.	Euphorbiaceae	coroncondo	Leaves, Roots	Decoction, Maceration	I/E	F, F*, M, N	5 (2 Pepel, 1 Fula, 1 Mandinga, 1 Mandjaco)		
*Jatropha curcas* L.	Euphorbiaceae	pulga, tao tao	Fruits, Latex, Leaves, Roots, Seeds	Decoction, Extraction, Ground, Maceration, Oil	I/E	B, C, D, INFI, M, MET, OR, Q	16 (4 Balanta, 4 Felupe, 4 Mancanha, 2 Mandjaco, 1 Bijagos, 1 Pepel)	X	
*Manihot esculenta* Crantz	Euphorbiaceae	mandioca	Leaves	Ground	I	B	1 (Balanta)		Food
*Arachis hypogaea* L.	Fabaceae	mancara	Seeds	Ground	I	B	2 (Mancanha)		Food
*Cassia sieberiana* DC.	Fabaceae	canafistra, utam	Leaves, Roots	Decoction, Maceration	I	B, F, J, MET	4 (3 Pepel, 1 Mandjaco)	X	
*Cajanus cajan* (L.) Huth	Fabaceae	fisson conco	Leaves	Decoction	I/E	F*	1 (Pepel)		
*Mucuna pruriens* (L.) DC.	Fabaceae	ng’hanhe, ghanhima	Seeds	Extraction	E	AP, C	2 (Balanta)		
*Piliostigma thonningii* (Schumach.) Milne-Redh.	Fabaceae	unha di vaca, pata-di-vaca, fara, pano-di-cancuran)	Fruits	Ground	I	E	1 (Pepel)	X	
*Cordyla pinnata* (Lepr. ex A.Rich.) Milne- Redh.	Fabaceae	psila, capsila	Bark	Decoction	I	A, J	3 (Balanta)	X	Food
*Dialium guineense* Willd.	Fabaceae	po di veludo,	Fruits	Maceration, Syrup	I	E, FOR, F*	5 (2 Pepel, 1 Balanta, 1 Felupe, 1 Mancanha)	X	Food
*Erythrina senegalensis* DC	Fabaceae	m’zisse, dolin	Branches, Seeds	Ground	E	R	1 (Balanta)	X	
*Erythrophleum suaveolens* (Guill. and Perr.) Brenan	Fabaceae	unconé, manconi	Bark	Ground	E	D	1 (Pepel)		
*Faidherbia albida* (Delile) A.Chev.	Fabaceae	acacia, fidida branca	Branches, Leaves	Decoction	I/E	A, F, F*	6 (3 Pepel, 1 Balanta, 1 Fula, 1 Mandjaco)		
*Parkia biglobosa* (Jacq.) G.Don	Fabaceae	foroba	Seeds	Juice, Maceration	I	D, F, F*, FOR, INFI	18 (10 Balanta, 5 Pepel, 2 Mancanha, 1 Mandjaco,)	X	Food
*Pterocarpus erinaceus* Lam. ex Poir.	Fabaceae	po di sangue	Bark	Maceration	I	D, E, F, J, N	11 (4 Balanta, 4 Bijago, 3 Pepel)	X	
*Senna occidentalis* (L.) Link	Fabaceae	padja santa, m’bete	Leaves, Roots, Seeds	Decoction, Maceration	I/E	A, B, C, E, F, F*, INFE, L, MET, N	24 (12 Balanta, 8 Mandjaco, 3 Pepel, 1 Mancanha)	X	
*Tamarindus indica* L.	Fabaceae	tambarina	Bark, Fruits, Leaves	Chewed, Decoction,	I	A, F, INFE, INFI, O	9 (4 Mancanha, 3 Balanta, 1 Bijago, 1 Felupe)		Food
*Vigna unguiculata* (L.) Walp.	Fabaceae	fisson mancanha	Seeds	Decoction	I	J	1 (Pepel)	X	Food
*Icacina oliviformis* (Poir.) J.Raynal	Icacinaceae	manganas, unas, foie	Bark, Leaves, Roots, Seeds	Chewed, Decoction, Ground	I	B, D, G, O	6 (4 Balanta, 1 Felupe, 1 Pepel)	X	Food
*Mentha sp.*	Lamiaceae	ortelon, nana, menta	Leaves	Decoction	I	MET	2 (1 Mancanha, 1 Pepel)		
*Cassytha filiformis* L.	Lauraceae	ridia di santchu	Branches	Decoction	I	E	1 (Pepel)	X	
*Laurus nobilis* L.	Lauraceae	padja lur	Leaves	Decoction	I	C, E	3 (1 Felupe, 1 Mancanha, 1 Pepel)		
*Abelmoschus esculentus* (L) Moench.	Malvaceae	candja	Fruits, Leaves	Decoction, Ground	I/E	D, MET	2 (1 Balanta, 1 Mancanha)		
*Adansonia digitata* L.	Malvaceae	cabacera	Bark, Fruits, Leaves, Seeds	Decoction, Maceration	I/E	AP, C, D, E, F, F*, FOR, J, N	13 (5 Pepel, 3 Balanta, 3 Mancanha, 2 Bijagos)	X	Food
*Ceiba pentandra* (L.) Gaertn	Malvaceae	polom	Fruits	Decoction, Ground	I/E	D	2 (Balanta)	X	Cosm
*Cola cordifolia (Cav.)* R.Br.	Malvaceae	mandjandja	Leaves	Decoction	I	MET	1 (Mandjaco)	X	Food
*Gossypium hirsutum* L.	Malvaceae	algadon	Leaves	Decoction	I	E	1 (Mancanha)		
*Hibiscus sabdariffa L.*	Malvaceae	badjigui, baguitchi, carcadè, ondjo	Flowers, Leaves	Decoction, Infusion	I	B, J, MET, N	4 (2 Balanta, 2 Pepel)		
*Carapa procera* DC	Meliaceae	citi malgos, mpalack	Seeds	Decoction, Oil	I/E	A, C, E, OR	13 (5 Pepel, 3 Balanta, 3 Mancanha, 2 Mandjaco)	X	
*Khaya senegalensis* (Desv.) A.Juss.	Meliaceae	po di bissilon, bissilon, iagu di polon	Bark	Decoction, Maceration	I	C, D, E, F, J, O	7 (2 Balanta, 2 Mancanha, 1 Bijago, 1 Felupe, 1 Mandjaco)	X	
*Moringa oleifera* Lam.	Moringaceae	nené badadji	Bark, Branches, Leaves	Decoction, Ground, Maceration	I, E	B, F, FOR, INFI, J, L, MET, Q	11 (4 Balanta, 2 Fula, 2 Pepel, 1 Felupe, 1 Mancanha)	X	Food
*Musa* × *paradisiaca* L.	Musaceae	banana	Leaves	Decoction, Smoked	I	B, E, F*, H, J, MET, N	11 (5 Mancanha, 3 Balanta, 1 Fula, 1 Mandjaco,1 Pepel)		Food
*Musa acuminata* Colla subsp. *acuminata*	Musaceae	banana	Leaves	Decoction	I	F, INFI	2 (Balanta)		Food
*Eucaliptus globulus* Labill.	Myrtaceae	eucalipto, hotubutel	Leaves	Decoction	E	E, F, F*	6 (4 Balanta, 2 Mancanha)		
*Psydium guajava* L.	Myrtaceae	goiaba	Leaves	Chewed, Decoction	I	C	6 (2 Balanta, 2 Mancanha, 1 Fula, 1 Pepel)		
*Sesamum radiatum* Schumach. and Thonn.	Pedaliaceae	lalocaminho	Flowers	Ground	E	AP	1 (Pepel)		Food
*Oryza glarberrima* Steud.	Poaceae	arroz preto	Seeds	Decoction	I	F, INFI	4 (2 Bijagos, 1 Felupe, 1 Mancanha)		
*Oxithenanthera abysynica* (A.Rich.) Munro	Poaceae	cana di bambu	Leaves	Decoction	I/E	D, MET	2 (Pepel)		
*Zea mays* L.	Poaceae	midju bassil, mais	Flowers	Decoction	I	N, MET	2 (Balanta)		Food
*Morinda chrysorhiza* (Thonn.) DC.	Rubiaceae	bulungudjuba	Leaves	Decoction	I/E	B, D	2 (Mancanha)	X	
*Sarcocephalus latifolius* (Sm.) E. A. Bruce	Rubiaceae	madronha, theeh intukidi	Bark, Roots	Decoction, Maceration	I, I/E	A, B, C, J, O	18 (9 Balantas, 4 Mancanha, 3 Pepel, 2 Mandjaco)	X	Food
*Spermacoce verticillata* L.	Rubiaceae	djalonca di caminho	Leaves	Decoction	I	D, MET	2 (Mancanha)	X	
*Citrus aurantifolia* (Christm.) Swingle Osbeck	Rutaceae	limon	Fruits, Leaves	Decoction, Juice	I	E, F, F*, J, L	12 (5 Mancanha, 3 Balanta, 3 Pepel, 1 Mandjaco)	X	Food
*Citrus sinensis* (L.) Osbeck	Rutaceae	laranja	Bark	Decoction	I	E	1 (Mancanha)		
*Vitellaria paradoxa* C.F.Gaertn.	Sapotacea	bambatulu, karitè	Seeds	Extraction	E		2 (1 Mandjaco, 1 Pepel)		Cosm
*Capsicum annuum* L.	Solanaceae	malgueta, idjodjo pket	Fruits, Leaves	Ground, Maceration, Poultice	E	A, D, E	5 (3 Pepel, 1 Bijagos, 1 Mandjaco)	X	Food
*Physalis angulata* L.	Solanaceae	tao tao	Roots	Decoction	I	E	1 (Mancanha)		
*Solanum anguivi* Lam.	Solanaceae	djagatu	Fruits	Decoction	I	Q	1 (Balanta)		
*Solanum lycopersicum* L.	Solanaceae	camate, tomate	Leaves	Fresh	E	D	1 (Felupe)		Food

Legend. **Disease categories**—A: Pains; AP: Anti-parasitic; B: Pregnancy, childbirth, breastfeeding, and diseases of the newborn; C: Intestinal problems; D: Skin inflammations, wounds, and burns; E: Cough and respiratory diseases; F: Fever and malaria (species indicated specifically for malaria, e.g., high fever associated with shaking chills, are marked with F*); FOR: General weakness; G: Stings, bites and poisoning; H: Mental and neurological disorders; INFE: External viral infections; INFI: Internal viral infections; J: Anemia and blood disorders; K: Male impotence; L: General infections; M: Diseases of the liver; MET: metabolic disorders; N: Diseases of the kidney; O: Other diseases; OR: Oral cavity diseases; P: Hemorrhoids; Q: Heart conditions; R: Bones and joints. **Use**—E: External; I: Internal.

**Table 2:** Values of RFC concerning the most cited medicinal plants as referred by informants.

Species	RFC
*Sarcocephalus latifolius* (Sm.) E.A.Bruce	0.286
*Senna occidentalis* (L.) Link	0.265
*Carica papaya* L.	0.245
*Jatropha curcas* L.	0.224
*Combretum micranthum* G.Don	0.184
*Terminalia macroptera* Guill. and Perr.	0.184
*Parkia biglobosa* (Jacq.) G.Don	0.184
*Carapa procera* DC.	0.184
*Elaeis guineensis* Jacq.	0.163
*Pterocarpus erinaceus* Lam. ex Poir.	0.163
*Adansonia digitata* L.	0.163
*Moringa oleifera* Lam.	0.163
*Citrus aurantifolia* (Christm.) Swingle	0.143
*Tamarindus indica* L.	0.143
*Guiera senegalensis* J.F.Gmel.	0.122
*Khaya senegalensis* (Desv.) A.Juss.	0.122
*Acacia albida* Del.	0.122
*Musa* × *paradisiaca* L.	0.122
*Psidium guajava* L.	0.122
*Anacardium occidentale* L.	0.102
*Mangifera indica* L.	0.102
*Dialium guineense* Willd.	0.102
*Icacina oliviformis* (Poir.) J.Raynal	0.102

**Table 3:** Several plants, and their main uses, that are regularly maintained by the majority of informants in Italy.

Vernacular Names	Species	Most Common Uses in Italy	Parts Used	Frequency of Use in Italy
cabacera	*Adansonia digitata* L.	As tonic and invigorating	Fruits/seeds	+++
nené badadji	*Moringa oleifera* Lam.	As invigorating	Leaves	++
badjigui	*Hibiscus sabdariffa* L.	Treatment of anemia and blood disorders	Flowers/fruits	+++
foroba	*Parkia biglobosa* (Jacq.) G.Don	As invigorating	Fruits/seeds	+++
candja	*Abelmoschus escuentus* (L) Moench.	Treatment of anemia	Flowers/fruits	+++
madronha	*Sarcocephalus latifolius* (Sm.) E. A. Bruc	Treatment of infant colic	Roots	+++
po di veludo	*Dialium guineense* Willd.	Juice as food supplement	Fruits/seeds	+++
citi malgos	*Carapa procera* DC	Treatment of infant colic; umbilical cord care in newborns	Seeds	+++
padja santa	*Senna occidentalis* (L.) Link	Juice against fever	Leaves	+
tambarina	*Tamarindus indica* L.	Treatment of internal infection with diarrhea	Fruits	+++
mango	*Mangifera indica* L.	Juice as tonic	Fruits	+++
goiaba	*Psydium guajava* L.	Treatment of diarrhea and intestinal problems	Leaves/fruits	+
cadju	*Anacardium occidentale* L.	Tonic	Seeds/juice	++
banana	*Musa* × *paradisiaca* L.	Leg swelling during pregnancy; itching caused by measles in children	Leaves	+
mandiple	*Spondias mombin* L.	Treatment of intestinal problems	Fruits	+
mandioca	*Manihot esculenta* Crantz	As tonic and food supplement	Leaves	++
papaya	*Carica papaya* L.	Treatment of malaria and as anti-parasitic	Fruits	+
limon	*Citrus aurantifolia* (Christm.) Swingle Osbeck	Treatment of respiratory diseases and fever	Fruits/leaves	+
laranja	*Citrus sinensis* (L.) Osbeck	Treatment of respiratory diseases	Fruits/leaves	+
algodon	*Gossypium hirsutum* L.	Treatment of respiratory diseases	Leaves	+

+++ high; ++ moderate; + low.

**Table 4:** Jaccard dissimilarity between the medicinal uses reported by Bissau-Guinean community of migrants living in northern Italy and reference information of native healers in Guinea-Bissau as in Catarino et al. [11].

Informants in the Italian Community				Vs. Whole Informants of the Italian Community	Vs. Whole Informants in Guinea-Bissau	Vs. Respective Ethnic Group in Guinea-Bissau
Ethnic Group	Males	Females	Total			
Whole Italian community	26	23	49		0.10	0.10
Balanta	12	9	21	0.01	0.11	0.11
Mancanha	1	5	6	0.42	0.48	0.44
Mandjaco	2	3	5	0.19	0.27	0.10
Pepel	4	5	9	0.08	0.17	0.18
Bijagos	2	1	3	0.67	0.70	0.70
Felupe	3	0	3	0.55	0.60	0.50
Fula	1	0	1	0.62	0.66	0.65

## Data Availability

All data supporting the results of this research are included within the article (see Table 1).
